# Embodied Cognition and the Direct Induction of Affect as a Compliment to Cognitive Behavioural Therapy †

**DOI:** 10.3390/bs8030029

**Published:** 2018-02-26

**Authors:** Tania Pietrzak, Christina Lohr, Beverly Jahn, Gernot Hauke

**Affiliations:** 1 School Psychology and Public Health, La Trobe University, Melbourne 3086, Australia; 2 Embodiment Resource Academy Europa (Munich), 80634 Munich, Germany; c.lohr@era-europa.com (C.L.); g.hauke@era-europa.com (G.H.); 3 Embodiment Resources Academy Europa (Leipzig), 04105 Leipzig, Germany; b.jahn@era-europa.com

**Keywords:** embodiment, CBT, interpersonal synchrony, therapeutic alliance, emotional regulation, emotional field, emotional mastery, affect experience, change process

## Abstract

We make the case for the possible integration of affect experience induced via embodiment techniques with CBT for the treatment of emotional disorders in clinical settings. Theoretically we propose a possible integration of cognitive behavioural theory, neuroscience, embodied cognition and important processes of client change outcomes such as the therapeutic alliance to enhance client outcomes. We draw from evidence of bidirectional effects between embodiment modes of bottom-up (sensory-motor simulations giving rise to important basis of knowledge) and top-down (abstract mental representations of knowledge) processes such as CBT in psychotherapy. The paper first describes the dominance and success of CBT for the treatment of a wide range of clinical disorders. Some limitations of CBT, particularly for depression are also outlined. There is a growing body of evidence for the added value of experiential affect-focused interventions combined with CBT. Evidence for the embodied model of cognition and emotion is reviewed. Advantages of embodiment is highlighted as a complimentary process model to deepen the intensity and valence of affective experience. It is suggested that an integrated embodiment approach with CBT enhances outcomes across a wide range of emotional disorders. A description of our embodiment method integrated with CBT for inducing affective experience, emotional regulation, acceptance of unwanted emotions and emotional mastery is given. Finally, the paper highlights the importance of the therapeutic alliance as a critical component of the change process. The paper ends with a case study highlighting some clinical strategies that may aid the therapist to integrate embodiment techniques in CBT that can further explore in future research on affective experience in CBT for a wider range of clinical disorders.

## 1. Introduction

Cognitive Therapy is an umbrella term to encompass a wide range of therapies aimed at changing cognitions and has been the dominant psychological treatment paradigm since the 1950s [1]. It has centred around Beck’s Cognitive Behaviour Therapy Model (CBT) [2] and Albert Ellis’s Rational Emotive Therapy (RET) [3]. Beck’s cognitive model has enjoyed much success, with numerous studies demonstrating the effectiveness of CBT for the treatment of anxiety through exposure protocols and inhibitory learning [4] and for depression [5]. CBT processes linked to the successful treatment of depression include cognitive (e.g., changing cognitions) and behavioural changes (e.g., inhibitory learning, problem solving skills, activity scheduling) and the therapeutic alliance [6]. CBT for the most part, assumes that maladaptive and distorted beliefs underlie symptoms and dysfunctional affect and behaviour and that these beliefs are behaviourally reinforced. CBT involves attention to a set of dysfunctional automatic thoughts and deeply ingrained belief systems (often referred to as schemas), along with learning and practicing new, non-maladaptive behaviours through cognitive disputation techniques.

Older meta-analytic reviews [5] of CBT treatment outcomes of a wide range of psychiatric disorders found large effect sizes for unipolar depression, generalized anxiety disorder, panic disorder, social phobia, posttraumatic stress disorder, childhood depressive and anxiety disorders. Recent meta-analytic studies have demonstrated the benefits of CBT for psychosis [7,8,9,10,11,12], eating disorders [13], personality disorders [14] and substance disorders [15,16,17]. 

### 1.1. Limitations of Cognitive Therapies

Whilst CBT remains a dominant paradigm and represents one of the most effective ‘evidence-based’ approaches in reducing mental health disorders- particularly for anxiety disorders, there are some limitations. These include: (a) different methodologies used across the various cognitive therapies in randomized controlled studies; (b) non-responders to CBT particularly for chronic depression and some personality disorders; and (c) the central theory (sandwich model-see below) from which CBT originates, with emotions seen as part of the response ‘bundle,’ has been questioned in light of recent bottom-up neurobiological and experimental findings; (d) recent research also suggests that affective experience is emerging as an important outcome treatment variable; (e) the possible need for integration of CBT with emotionally focused treatment approaches. Each of these limitations are addressed in more detail below.

### 1.2. Different Methodologies

There is variation and complexity across the various ‘cognitive’ models and how the intervention strategies are implemented. There are differences in intervention strategies between RET, CBT, Schema Therapy [18] and Mindfulness based CBT [19,20,21] even though they all fall under the umbrella of ‘cognitive’ therapy. To highlight this variation in cognitive therapy, Albert Ellis [22] highlighted the differences between RET and CBT. Cognitively, RET has a pronounced humanistic existential philosophic emphasis whereas CBT does not. Ellis also argued that RET recognizes the value of cognitive distraction, discourages problem solving that is not accompanied by changes in clients’ basic belief system and emphasizes secondary as well as primary symptoms of emotional disturbance. Emotively, compared to CBT, RET emphasizes methods of working directly with emotions and sometimes encourages forceful emotive interventions. Behaviourally, it encourages punishment as well as reinforcement and is partial to in vivo desensitization and flooding [22]. 

Compared to CBT and RET, mindfulness therapy endorses the observation and acceptance of all emotions and all thoughts (wanted and unwanted), encourages clients to pay attention to and be in sensory rather than thinking modes. Cognitive disputations and restructuring thoughts are not overly emphasized. Schema therapy combines CBT, gestalt, imagery and other behavioural techniques to help client’s gain insight into their problematic and unhealthy emotional and behavioural survival modes learnt from childhood (schemas). Clients learn how to break old patterns and rebuild their healthy adult side. Schema therapy goes beyond anxiety and depression and works to rectify deep emotional disturbances held since childhood and to change long term patterns. Whilst schema therapy includes elements of CBT, these elements are often practiced later. Whereas in CBT there is a strong emphasis on helping clients to recognise automatic thoughts and how these automatic thoughts make clients feel and behave.

The National Institute for Health and Clinical Excellence (NICE) endorses Cognitive Therapy because of its strong research base, however there are many disputes about the different methodologies used in randomized controlled trials across the different cognitive therapies. Whilst the NICE guidelines support CBT, they do not support the superiority of CBT over all other interventions [23].

### 1.3. Non-Responders to CBT

There is emerging evidence that in CBT treatment for depressive disorders, a significant proportion of depressed clients or those with a personality disorder do not benefit [24] and there are high drop-out rates [25]. The average improvement among chronically and pervasively depressed CBT clients is between 20–50% [26]. CBT for the treatment of the personality disorders is described in detail by Beck et al. in their book ‘Cognitive Therapy of Personality Disorders’ [14]. However, because persistent dysfunctional belief systems in clients with personality disorders are usually embedded into their usual cognitive organization, substantial time and effort are required to produce lasting change. Therefore, modifications of standard CBT approaches have emerged to treat personality disorders. For example, schema-focused cognitive therapy or dialectical behaviour therapy are often recommended as the treatment of choice in treating certain features typical of the personality disorders. However, other than dialectical behaviour therapy that has a strong evidence base [27,28,29,30,31], these modifications have not been rigorously studied. In sum, most of the CBT studies in the treatment of personality disorders involved dialectical behaviour therapy and even then, replication studies by other groups are needed to confirm the validity and generalizability of these findings.

### 1.4. Sandwich Model of CBT 

The sandwich model of CBT [32] is reflected in the direction of effect as represented by the behavioural SORKC (Stimulus, Organism, Reaction/behaviour, Contingency, Consequence) model that describes five determining factors as a basis of learning processes. The sandwich model serves as an important basis for behavioural therapy case conceptualization: a certain stimulus leads to a mental experience that results in a felt emotion with accompanying body sensations and certain physical actions. Barsalou suggests that in the sandwich model, the body is viewed as an ‘output unit’ [32]. For example, when a loss of job (S) is interpreted as ‘terrible and awful with catastrophic consequences’ (cognition) leads to a collapsed body posture (R) and withdrawal and lack of help seeking behaviour to find a new job (K, C). The case conceptualization frame is important as it gives the therapist a solid experience in analysing stimuli, the presence of automatic thoughts and schemata, as well as systematic logical errors in thinking which maintain negative emotional states and problematic consequences. CBT aims to teach clients to notice and change negative thoughts which maintain the negative emotion and problematic behavioural outcomes. CBT is a ‘top-down’ approach that generally assumes that the content of thoughts is the key causal variable in maintaining and exacerbating negative mood. Therefore, intervention mainly involves the therapist helping the client to change their cognitions or the degree of belief of their thoughts [33]. Having said this, Aaron Beck and other CBT theorists and practitioners have maintained that although dysfunctional thoughts is a major potential pathway leading to emotional dysregulation, it is possible that emotions and physiological states and sensations can first be felt and then later appraised albeit incorrectly. This incorrect appraisal can lead to a type of cognitive distortion known as emotional reasoning [34]. Chapter 4 in Tolin’s ‘Doing CBT’ states “It’s a mistake to conclude that all emotions are caused by thoughts. There are other roads to emotion” [35]. Similarly, Zajonc [36] claimed that there are circumstances under which affect precedes cognition and that affective arousal can exist without prior cognitive appraisal.

### 1.5. Limitations of the Sandwich Model

Motivational systems and attachment processes reflecting various developmental needs and stages are not engaged well by CBT approaches [37,38,39]. Moreover, there are some theoretical reasons to question the cognitive paradigm that views cognition in the sandwich model as a processing of abstract symbols according to rules in a central processor like a computer that transforms inputs into outputs. In this view, it remains unclear how cognition interfaces with perception and action [40,41]. CBT [42] developed from cognitive science, where knowledge of the world occurs within a conceptual system that contains semantic information about a category. Knowledge is built up by links between categories [33]. According to the cognitivist tradition, this knowledge supports and controls other cognitive functions such as language, thinking, perception and memory. Thus, knowledge is abstracted from sensory/perceptual systems that originally encoded them, therefore concepts take on an ‘amodal quality’. Perceptual information is lost or transduced when the concept is ‘abstracted’ from the sensory modalities that were originally involved in perception [33]. Therefore, cognition is viewed as independent of the medium, independent of the body, creating the mind-body divide (dual aspect theory) [43,44].

However, neuroscience with its activation of brain systems such as mirror neurons responsible for emotional attunement, is confronting CBT with some major processing systems that cannot be classified as just ‘cognitive’ [45]. Furthermore, according to significant findings in embodiment research [46,47,48] the reverse direction of effect also applies in the sandwich model. The bidirectionality principle of embodiment theory suggests that movement and body interaction are comprised of basic sense-making with afferent feedback loops to emotional and cognitive levels [49,50]. So, emotionally focused treatment that successfully reduces negative mood states via direct induction of emotions, also reduces the frequency, intensity and degree of belief of negative thoughts and schemas [33,51].

A final point is the existence of some gaps in empirical research about the potential added value of CBT going beyond cognitive and behavioural change to integrate emotionally focused direct induction of both positive and negative affect experiences [52,53]. Exposure tasks in CBT have traditionally focused on inhibiting avoidance/escape responses related to high levels of pathological anxiety and not enough emphasis has been given to the direct induction of a variety of both positively and negatively valanced emotions (e.g., anger, sadness, shame, guilt, pride, tenderness, happiness and eroticism). In the general practice of CBT, direct induction of a variety of different emotions appears to be the exception rather than the rule. We have known for a long time now that activation and escalation of fear within the framework of behavioural therapy exposure treatments can be essential predicators of the success of therapy [54]. Within the context of exposure therapy, new developments regarding inhibitory learning refer to the notion that fear associations are not removed during extinction but rather remain intact as new learning about the feared stimulus occurs [55]. At this point the importance of acceptance strategies was highlighted [56]. Although modern concepts come into play, research on activation of emotions different to anxiety remains raw in the explicit context of CBT. 

New research developments highlighting inhibition learning by Watson and Bedard [57], compared the therapy results of CBT clients with those who had completed an experiential therapy. The CBT client group showed a significantly poorer outcome combined with a substantially lower emotional processing depth. Inspired by these promising results on the added value of including some aspect in therapy of deepening emotional processing, in recent years researchers have been focusing more on the significance of emotional processes as outcome variables. 

### 1.6. Affect Experience as an Important Treatment Outcome Variable

New research [33,52,53] suggests that is important to consider the clients’ full range of affect experience (AE), as a changeable in-session process that may relate to CBT treatment outcome. Samoilov and Goldfried [58] even went as far as to say that AE has remained “terra incognita” in both research and clinical practice for a long time now and that activation and escalation of fear within the framework of behavioural therapy exposure treatments can be essential predictors of the success of therapy [54].

For CBT treatment outcomes, there exists increasing evidence that in-session AE is an important change variable in the treatment of PTSD [59], social anxiety disorder [60], panic disorder [61], phobias [62], cluster C personality disorders [63] and chronic fatigue [64]. Furthermore, AfjesVanDorn and Barber [52] highlight the growing research for experiential affect-focused interventions developed as adjunct to CBT (e.g., Exposure-based Cognitive Therapy for depression; [65]) and evidence based third-wave CBT approaches, further delineating the importance of AE and the associated cognitive processing of these emotional experiences in CBT treatment. However, what remains lacking in the exposure protocols associated with CBT treatment is the lack of full exploration of the network of all emotions associated with an affective disorder. For example, rather than solely focusing on those affects related to the diagnosis of depression (e.g., sadness, guilt), AE is able to capture the full range of affects of basic emotional systems- such as anger, anxiety, jealousy, shame, pride and tenderness [66].

Meta-analytic outcomes reviewed by Aafjes Van Doorn & Barber showed that whilst the deeper the emotional exposure process the greater the symptom reduction, the specific effects of AE depended on whether it included a cognitive element and the valence of the AE. They found across the 5 regression analysis studies, that the higher AE levels were related to lower symptom levels when AE included an aspect of cognitive processing and/or when it measured AE with a positive valence, such as feeling excited or happy [67]. The predicted symptom improvement of AE with cognitive processing that also included positive valences [68,69,70,71] suggests that AE might lead to change in CBT for depression. These findings are consistent with the theoretical models of Epstein [72], Barnard and Teasdale [73] and Hayes and Harris [74] that forms the foundation for the Exposure-Based Cognitive Therapy model for depression [75]. 

Furthermore, Gjelsvik and Lovric [33] reviewed recent experimental and clinical studies that manipulated the level of abstraction in therapy as well as studies that increased ‘embodied processing’ in treatment. Embodied processing according to the authors suggests that “a necessary ground for cognition is the way our sensory-motor capacities shape and constrain our interaction with the world.” Embodied theories posit that meaning, previously understood in terms of the language of thought, or as abstract symbols, is much more closely intertwined with (and constrained by) our bodily experience in the world than such abstract symbols allow. Therefore, meaning is not something that is removed from our bodily experience but is instead intertwined with it to function effectively [33,40].

Gjelsvik and Lovric found some evidence to suggest that emotional disorders can arise as a result of failed emotional simulation processes. The authors even went so far as to hypothesize that over-use of language-based, abstract processing could possibly maintain psychopathology. To be more specific, the authors propose that an over-reliance on abstract, language-based processing to reduce negative affect can sometimes be unhelpful if there is a discrepancy between one’s current state and the expectation to be happy. Thus, checking the degree of discrepancy and finding a mismatch can increase distress, so that the goal of being happy and feeling better is further away [33]. Experimental studies manipulated the subject’s level of abstraction in an intervention during a failure task and demonstrated that concrete experiential self-focus (e.g., how the body feels relating to the experience) increased memory specificity, enhanced social problem solving and reduced emotional disturbance in depressed subjects compared to those that processed a failure task in a more abstract way (e.g., thinking about causes, meanings, consequences of the experience) [76,77].

Starting from the classical CBT perspective, Gjelsvik et al. [33] suggest that an integrated model of bottom-up and top-down modes is important to consider when working with emotional disorders. Top-down processes can rely on over-abstract processing and fail to move down from abstract to more sensory-perceptual modes of cognition. This can result in the client remaining in conceptual realm, potentially weakening top-down control of emotion via language. The result can be excessive rumination and difficulty in retrieving specific autobiographical memories [33].

## 2. Integration of CBT with Emotionally Focused Treatment (EFT) Approaches

Independently of the respective therapy approaches, clinical research has found that emotional activation, its intensity and the processing of the emotional experience in therapy, are decisive for its success [57,78,79,80,81,82]. Furthermore, the majority of psychologists and trainees characterize their therapeutic approach as “eclectic/integrative” [40,83]. An alarmingly shrinking number of psychologists (as low as 2%) use a pure therapeutical orientation [41,84]. A more dominant emerging trend is shifting towards assimilative integration in psychotherapy practice [50,85].

EFT is a process-oriented method aiming to improve a client’s ability to constructively deal with emotions [45]. Although various types of emotional processing exist between the different types of EFTs requiring different interventions, there are also important common factors between them. These include the distinction between primary versus secondary and adaptive versus maladaptive emotions. EFT assumes that a client’s problems are often related to an inability to gain insight and understand their own emotions and thus an inability to derive appropriate responses. EFT also postulates that clients often have an inability to expose themselves to threatening or painful emotions, even though there may be personal growth as a result. 

Methodologically speaking the therapy procedure is guided by indicators for problems in emotional processing but also for a client’s readiness to face their emotional problems. EFT has acquired some scientific grounding in several empirical studies [46,86] and is considered an empirically validated treatment for depression, couples’ therapy as well as other disorders [45,47,48,51,87,88,89,90]. In recent years, the trans-diagnostic topic of emotion regulation has become increasingly important. The prominent approach by Gross [91,92] is closely adjacent to the CBT framework. It has been shown that independently of disorder-specific therapy concepts or specific mental disorders, emotion-related interventions increase the efficiency of psychotherapy and especially CBT-interventions [93,94]. Especially the working group around David Barlow [95] emphasize that commonalities in aetiology and latent structure among emotional disorders supersede their differences.

Barlow et al. suggest the possibility of distilling a set of psychological procedures that would comprise a unified intervention for emotional disorders. Based on relevant theory and data they identify three fundamental therapeutic components relevant to the treatment of emotional disorders generally. These three components include (a) altering antecedent cognitive reappraisals; (b) preventing emotional avoidance; and (c) facilitating action tendencies not associated with the emotion that is dysregulated. This treatment takes place in the context of provoking emotional expression (emotional exposure) through situational, internal and somatic (interoceptive) cues. Under the influence of findings in the context of Acceptance and Commitment Therapy, Gratz and Roemer [96] propose a conceptualization of emotion regulation, which refers not only to the modulation of emotional arousal but explicitly to the awareness, understanding and acceptance of emotions, as well as the ability to act according to the emotional state.

The prerequisite of being successful in this point is the ability to discriminate the full range of different emotions and to understand their meanings. Both Gross’s [97] extended process model and his conceptual relatives believe that emotion regulation is driven by mental representations of goals and scoring systems. This more classical view refers to “static” representations and does not explain the dynamic nature of emotion regulation that results from our interaction with changing environments [98]. The embodiment approach with its term embodied cognition [32] also tries to take this dynamic into account. Cognition relies on physical states and physical action in this definition. The interaction of humans with their environment happens through the body. Emotion regulation is thus embodied and situated and it does not just happen in our heads [98]. This may give an additional argument for using bottom-oriented embodiment techniques. A recent randomised controlled trial by Babl et al. [53] is currently under-way that is examining the effectiveness of integrating components of EFT into an integrative form of CBT in a way that is replicating integrative practice common place in psychological therapy today. The aims of the study are to understand whether the emotionally focused integrative approach combined with CBT is more efficacious than treatment as usual (CBT alone) and to clarify the possible role of emotional processing as a mediator of therapy outcome. The project is original as it specifically examines whether EFT adds additional value to CBT. Clearly there is a current trend in research which substantiates the role of EFT which has already acquired some empirical validation across a range of emotional disorders [48,89].

Another very important reason to use an integrative psychotherapeutic framework is that not all psychotherapy clients, with their presenting problems, fit into a CBT treatment plan. Trying to ‘fit’ a client’s needs within a fixed traditional theoretical approach may not be optimal for every client [89]. Babl et al. go on further to outline that some clients suit a CBT treatment plan and others need different approaches for different problems (e.g., anxiety versus personality disorder). Furthermore, Babl et al. highlight there may be a change of needs for the client over time and not all relevant problems may be disclosed to the therapist at the beginning of the therapeutic process. Therefore, a psychotherapeutic approach is wise to be adaptable to the client’s needs and abilities as reflected in a case formulation [23]. To achieve this goal, it is optimal that for all change factors relevant to therapy outcomes, a sufficient range of interventions and concepts upon which they are based is available and empirically studied. 

## 3. The Embodied Model of Cognition and Emotion

Embodied approaches are based on the sensorimotor coupling between an organism and its environment [49] and have been evolving over the past 20 years. Embodiment theory takes the position that cognitive processes are not possible without the direct participation of the body because it has direct influences on cognitive, motivational and emotional processes through its posture, facial expressions, gestures and direction of movement [99].

Although there are different versions of embodiment theory from the moderate end [100,101] to the radical end [102]—all embodiment approaches have the idea that psychological processes are influenced by morphology, sensory systems, motor systems and emotions [103]. Emotions are viewed not just as categories we think about but involve bodily changes that have strong effects on cognition and action [104]. Barsalou [105], after reviewing a large number of studies, concluded that knowledge involves the activation of a ‘simulation process’ as if interacting with the world. Thus, via simulation, knowledge is viewed to be stored as re-enactments of perceptual, motor and introspective states acquired during interaction with objects, people and our own bodies. A simulation can generate a ‘feeling tone’ as well as partial or complete reactivation of an emotion and body sensations that became associated with the original experience [33]. 

Fuchs [43] identifies three cycles of embodiment as: (a) self-regulation involving a basic affective sense of self; (b) sensorimotor coupling between a person and their environment; and (c) intersubjective interaction or ‘intercorporeality’ as the basis of intentional cooperation, joint attention and verbal communication. Gallese’s [106,107] important research on mirror neurons supports the relationship between perception and action in social cognition through a tight functional coupling or interpersonal synchrony between actions produced by the self and actions perceived in others. This also applies to emotional coupling and body resonance. Embodied cognition qualifies as a bottom-up process and suggests that: (a) cognition and emotion are inherently interlinked with physical states and action; (b) cognition is based on intermodal feedback loops; (c) cognition and emotion can be based on simulation. Evidence drawn from a number of studies of behavioural, neurophysiological, neuroimaging and lesion studies [44,49,108] support the idea that cognition and emotion depend on the body and its interaction with the world. 

### Review of the Research in Support of Embodiment Techniques in Psychotherapy

Many studies have demonstrated the bi-directional link between nonverbal behaviour and accompanying body postures and human thought and feeling [109,110,111,112]. There are currently over 33 published studies on the positive effects of expansive (versus contractive) body postures on increasing subjective feelings of power, pain, hunger and romantic attractiveness [113,114]. In their original study, Carney et al. [115] also found an embodiment effect on increasing risk taking, as well as a biological effect of increasing the aggressive hormone testosterone and in reducing the stress hormone cortisol. Replication studies since 2010 however found no effect for risk taking or the same biological effects. Thus, the current position in the literature is that the postural effects of embodiment are bound by social context, culture and the participants awareness or not of the hypothesis [113].

Aside from the effects of expansive versus contracted body posture on felt power, several recent studies have begun to look at the effects of postural abnormalities and the effect on a range of different emotions and vice versa. For example, Price and Harmon-Jones [116] explored the influence of manipulated facial and bodily states on emotive responses. They found that body expressions play a pivotal role in the emotive experiences of people. Mondloch et al. [117] found that the accuracy and speed with which people identify emotional facial expressions is influenced by body postures. Research by Gross and John [118] and Joorman and Gotlib [119] showed that subjects who consciously expressed positive emotion with their facial expressions and body were able to counteract attentional focus on negative stimuli and their appraisal associated with depression. Subjects were more able to perform adaptive, mood improving cognitive reappraisals of ambiguous situations and challenges from deliberately induced positive emotions and accompanying body postures. This is a powerful example of an intervention that explicitly tried to change the level at which the negative material was processed, rather than trying to change the content of such material. In other words, without first changing the structure of thinking but instead engaging the client in bottom-up processes, the client’s attention to the body in relation to the event can generate a new felt experience and decentralize the client from their thinking. In essence, the body can give rise to new ways of thinking without the direct challenge of thought content as the client’s body has changed the way they relate to problem from the inside.

## 4. A unifying Perspective for Clinical Psychology: Integration of Bottom-Up and Top-Down Modes

Psychotherapy to date has been predominantly a talking therapy relying on cortical ‘top-down’ mechanisms. Top-down oriented psychotherapies such as CBT have the following characteristics [99]:

- Seek lingual expression for an experience, provide interpretation and re-interpretation of experiences.
- Identify and examine belief sets, compare, relativize and communicate what is experienced.
- Elaborate problem solutions, targets, plans and interim steps and the timing of these.
- Have a time frame perspective covering past experiences into future experiences.

Bottom-up therapies using embodiment techniques take the opposite course with the following characteristics [99]:

- Focus is placed on sensory and physical perceptions and impulses, movements of the whole body and parts of the body in space.
- Clients focus their attention and observe their body processes to gain access to the roots of their emotional experiences, to their automatic impulses and pre-lingual processes.
- Sensory motor input is induced by probing, tensing, moving, conscious breathing in order to place automated processes and categorizations into conscious awareness.
- Time perspective focuses on the ‘here and now,’ thus providing a chance of escaping the ‘memory trance,’ resisting automatisms and trying out alternatives.

Taking into consideration the bidirectional effects, one can conceptualize a unifying perspective for psychology that integrates the two modes of bottom-up and top-down. From this perspective, there is a circular and reciprocal influence of subjective, intersubjective and physiological factors on each other. To quote Fuchs [43], “The brain both reflects and causes alterations in the relationships with the body, the self and the world.”

Top-down therapies such as CBT have demonstrated evidence for reducing the pathology of clinical emotional disorders by changing the client’s implicit relational patterns, thoughts and behaviour with recent studies also showing functional and structural changes in the brains [43]. Therefore, it is prudent to embrace the effective top-down options of treatment. However, embodiment techniques offer simple, fast and significant effects and a new understanding that explicitly changes the level at which negative material is processed. Therefore, it makes sense to integrate the two approaches.

A satisfactory account of psychological interventions going into the future would be wise to consider both the embodiment perspective and the role of abstract conceptual thought to better understand the interplay between analogical representations that actively utilize the perceptual, somatosensory and motor resources and conceptual resources such as language like symbols. There have been some attempts to integrate the two approaches. For example, mindfulness based cognitive therapy (MBCT) [33] has the client shift from abstract language-based intervention to a concrete, embodied and experiential mode of processing. Results showed that MCBT significantly reduced risk of relapse of depression in the most vulnerable clients by 43% even though they did not find an overall effect of MBCT compared to controls. Recent research reviewed thus far suggests that embodiment can be complementary to CBT [120]. But what exactly are embodiment techniques? 

### 4.1. Embodiment Techniques Defined

Embodiment techniques are a defined practical procedure and include the guidance for arbitrarily setting a particular combination of body-related features, e.g., expansive posture, direction of gaze, direction of movement, respiratory patterns etc. [99,121]. This is guided by modelling by the therapist and imitation by the client. Body-related sensations and impulses are carefully observed and described. Clients are supported in developing “embodied self-awareness” to describe their experiences simply and directly. Conceptual thinking is omitted as much as possible. Embodiment techniques use body-related features of specific emotions which we coin ‘emotion patterns’. With the aid of body postures and movements, mimicry, gesture and partial breathing patterns, specific patterns of distinct emotions can be triggered. In using embodiment techniques, distributive associative patterns including different brain areas are triggered. Following many learning episodes an increasingly entrenched associative network reflects the aggregate effects of neural processing distributed across these areas [122].

### 4.2. Integrated Psychotherapy Approach: A Type of Embodied CBT

**Figure 1:**
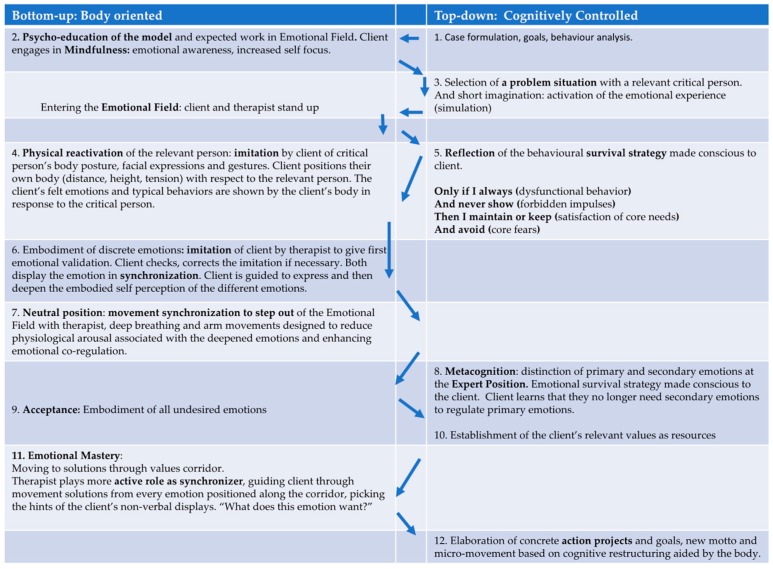
Overview of the switch between top-down and bottom-up orientation.

We propose an integrated switch model of psychotherapy for clinical disorders that combines the bottom-up processes of embodiment with the content-based top-down style of CBT (see Figure 1 below for the sequence of steps in the switch model). Theoretically, we propose to add a processing level to the abstract cognitive level of CBT based on the strengths of embodiment approaches and the limitations of CBT previously reviewed in this article. In our method, Step 1 one begins by adopting the CBT strategies of case formulation, past life history, goals for the future and behavioural analysis. In Step 2 the client switches to mindfulness awareness and learns how to do a body scan to observe discrete body sensations that accompany felt emotions. In Step 3 we switch again to the cognitive side by asking the client to think of a critical person in a critical situation where they felt the highest emotion. Here the client performs a short scenic imagination, also called imaginal exposure in CBT. Next, we switch back to the body orientated side in Step 4, introducing the client to embodiment techniques that deliberately move clients vertically down the hierarchy to more sensory-perceptual modes of cognition to use the client’s body to aid in the improvement of mood states and more positive cognitive reappraisals. This is done by having the client track their somatic markers and core affects in response to a specific critical person and situation.Specifically, our switch model contains elements bottom-up processing by: (1) directly addressing emotional exposure by induction of concrete problematic situations, (2) developing a body focus by exercising interoceptive awareness [123], (3) and then deepening this experience with the aid of embodiment techniques in the emotional field (see below). The arbitrary application of empirically validated body features (mimics, body posture, breathing patterns) supports the induction and immediate experience of specific emotions [122], (4) by that primary and secondary emotions are detectable and play an important role in dysfunctional emotion regulation and related emotional disorders [78,124,125].

### 4.3. Method of the Emotional Field in the Switch Model

**Figure 2:**
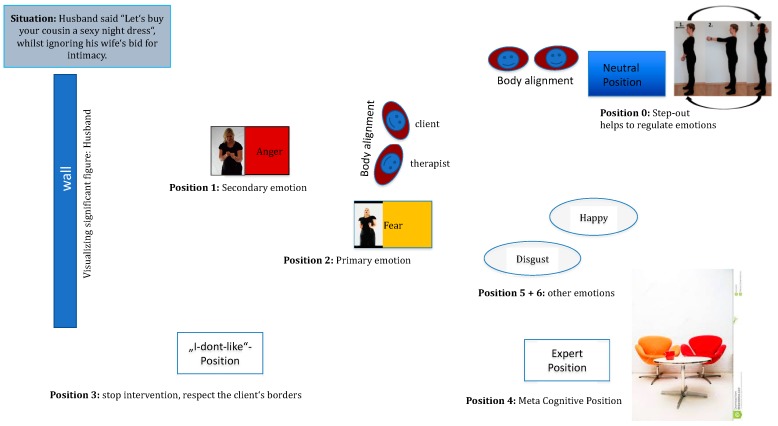
Hypothetical Emotional Field of a client.

The Emotional Field in Step 4 of the switch model, is an experiential domain mostly defined by putting the observation of the client’s body (nonverbal, displays, somatic markers) to the foreground (What does the body want to do?). It is shown below in Figure 2. By embodying cognitions and emotions, we propose several possible advantages that require empirical validation via randomized controlled studies. Theoretically, we propose a speeding up of the interpersonal process in the therapeutic relationship and an efficient processing of the clients’ concrete problems by deliberately using their body in a simulation. Furthermore, pre-lingual processes and emotions are made conscious in the Emotional Field, easily identifiable and given a lingual format to be reflected upon by the client. 

In Step 5 of Figure 1 we seek motivational clarification by helping the client elicit their embodied emotional survival strategy. The client’s insight into how their disorder originated and is maintained is an important factor for the effectiveness of client outcomes. One way of helping the client to achieve this is to use the survival strategy method emanating from Strategic Behavioural Therapy [45]. The survival strategy is the client’s dysfunctional cognitive-affective schema that in the interest of greatest possible need satisfaction, describes the various strategies the client employs for approach and avoidance in their social interactions. In the switch model, the client’s problems are processed in a clearly defined and concrete specific situation (when, where, with whom) with a focus on the client’s central needs related to their selected problem. Then the survival strategy that is to serve the satisfaction of the client’s need is elaborated by the therapist and written down to share with the client. By using an embodied simulation drawing from bottom-up input, the client does more than just report on the event after it happened but instead experiences the ‘event’ in the here and now. The simulation can be perceived as real by the client as it uses the same systems associated with the real event (neural networks that process information on space, time, action, affect and physical reactions).

Once the survival strategy has been obtained and reflected upon by the client sixth step (Figure 1) is to switch to specific embodiment techniques. These include imitation and synchronization as well as the deliberate manufacturing of client’s emotions in the Emotional Field-designed develop strong emotional regulation.

In the Emotional Field the communication is driven using the embodied-self (embodied self-perception) based on bodily sensations, immediate sensing and feeling in the present moment in reaction to the problematic person. Clients can also step out of the Emotional Field in the Neutral Position (Step 7, Figure 1) when their felt emotions are too under-regulated and physiological arousal is high. The step out procedure is designed for emotional containment and de-escalation through a deep breathing exercise in movement synchrony with the therapist with accompanying arm movements by cupping the hands and moving them in an upwards direction above the head and then back to resting position. 

In the Expert Position (Step 8, Figure 1) clients can engage in conceptual self -perception and reflection to identify and name the discrete emotions in the Emotional Field and the function of the emotions for self and interactional regulation. In the Expert Position, clients switch back to language, concepts and thought, using their rational and logical processes. Clients examine contents and begin reflecting and analysing the functions of their specific emotions and what solutions for emotional regulation were gained by the body. In Step 8 there is a careful exploration of the client’s immediate emotional experience before putting top-down statements forward. Direct induction, intensifying and experiencing a full range of emotions are in the foreground. Cognitive reappraisal (Step 12, Figure 1) is a by-product of emotional induction and is strengthened by tailored exercises in everyday life. Therefore, we propose that active cognitive restructuring of the client’s maladaptive thoughts, which is signature to the CBT method, is already given a helping hand by the pre-verbal information provided by the body being made conscious. More adaptive behavioural plans (Step 12) are formulated, relying on restructured thoughts, a new motto and a new body movement to support the plan in every-day life with the client’s critical people.

In order to represent the overall added value of the embodied CBT approach it is also useful to describe the embodiment techniques against the background of the general efficacy of psychotherapeutic factors such as mastery and resource activation [126] (Figure 1 Steps 10 and 11). The therapeutic alliance has also been strongly linked as a common factor essential for good client outcomes in psychotherapy. The therapeutic alliance will be discussed in the last section of the paper and is embedded into the entire switch model but is particularly enhanced via processes of imitation and movement synchronization (Figure 1, Steps 6 and 11).

### 4.4. Resource Activation and Emotional Mastery: Moving to Solutions

The client’s personal values serve as strong resources [99,127]. When values are just talked about and reflected on they become relatively abstract cognitive constructs, leading to the possibility of rumination rather than acting on them as a strengthening resource. The commitment to concrete goals, targets and behaviour plans is reinforced by the implementation of these values. Anchoring values in body postures and movements makes it possible to give values concrete form for the client and to use them as powerful companions like guiding stars for the client’s change process in everyday life. In our integrated model, we achieve this by developing analogously with the value attitude, a suitable body movement, picture and motto to reflect the body posture of the value (see Figure 1, Step 10). 

**Figure 3:**
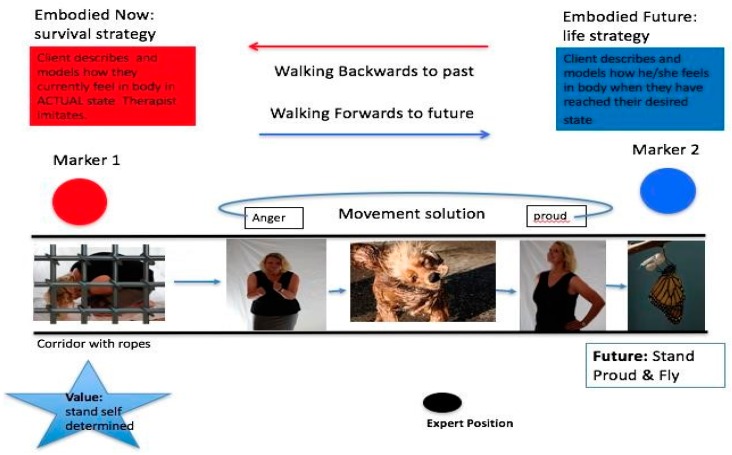
Emotional acceptance and mastery.

After establishing the relevant value as a resource, the therapist encourages the client to enter the passageway of the corridor (Figure 1, Step 11 and for more detail Figure 3 below). Next a defining path is set which has a start point (How body feels in actual state) and end point (How body feels when the client has reached their desired state). An image and motto is picked for the end state and the client shows a body posture or movement that is matching to the image. The therapist imitates the client’s embodied future. In the values corridor, a defining path of change is established. The client moves forwards through each emotion identified from the Emotional Field which is positioned along the corridor in a definable space with ropes and markers towards a movement solution. Here the therapist can change in following and taking the lead. The therapist usually plays a more active role in the corridor, acting as a synchronizer and guide. The therapist encourages the client along the corridor to embody each emotion and then to feel for a movement solution that will help the client get to their embodied future, picking up hints of the client’s nonverbal displays. In emotional mastery of our model, synchronization of the therapist’s movements with those of the client during a guided interaction in the values corridor is hypothesized to lead to prosocial consequences such as greater rapport, feelings of closeness between the client and the therapist [128,129]. 

Embodied cognition theory suggests that there is a link between bodily movements and cognitive functions [130]. Here the embodiment of a successful goal achievement path is one that leads forwards in space [131]. The corridor is defined by an initial state (see Marker 1, Figure 3) known as the client’s actual state as well as a desired state (see Marker 2, Figure 3) known as the end state. Forwards motions are associated with the concept of ‘future’ and reverse motions with the concept of ‘past’ [132]. Therefore, by asking the client to place themselves in the actual state and move towards the desired state, this simultaneously corresponds to the generation of future [133]. These tangible and concrete source concepts and the tension generated between the client’s actual and desired states is hypothesized to help the client to understand more clearly what the actual mental state looks like and how it differs from their desired end state. We also suggest that the client is more able to decide what is no longer tolerable in an actual situation and what they want to experience and be like in the future. The final step in the mastery phase switches back to the cognitive side (Figure 1, Step 12), where conceptual talk again emerges between the client and therapist to set concrete action plans and goals for homework. Here clients cognitively reappraise their thoughts on the problem situation and person and develop a new motto and movement to help them remember to implement the new behaviour plan.

### 4.5. Preliminary Support for the Switch Model of Embodied CBT

There is some preliminary data using single case study designs [99,121] and embodied CBT for single couples [134] and groups of couples [135] showing some promising results. For example, after 20 h of group couple embodied CBT intervention, the couple treatment group (compared to the control group) showed significant and meaningful increases in relationship satisfaction and empathy. Limitations of this preliminary research include only a limited number of studies, mostly qualitative data in case study design format. Another limitation for the group couple study [135] was that we could not be sure that the superior results for the treatment group were attributable to the integration of emotional embodiment techniques in CBT. It may have been that either CBT alone, or embodiment alone could have also produced such a good effect for the treatment group compared to the control group. Future research is needed in a randomized control format comparing CBT alone, embodiment alone and an emotionally focused integration of the two to determine the relative contributions of each approach to client outcomes.

## 5. Therapeutic Alliance

The therapeutic alliance is an important psychotherapeutic factor linked to efficacy in client outcomes across all methods of psychotherapy. However, in our integrated model we go one step further by viewing the strong development of the therapeutic relationship as an important embodiment technique. Our embodiment techniques of working with the therapeutic relationship is guided by the pioneering Interpersonal Synchrony (IS) model developed by Koole and Tschacher, grounded in neuroscience [136]. Koole and Tschacher propose that the therapeutic alliance develops unconsciously and spontaneously as a consequence of interpersonal synchrony (also known as inter-brain coupling) between the therapist and the client. The strength of the therapeutic alliance is an important precursor for the later effectiveness of intervention techniques to develop their full effects [132,137]. Furthermore, interpersonal synchrony between the client and therapist fosters the therapeutic alliance which can act as a tool for adaptive emotional coregulation between them. The client’s self-disclosure [138] and the wish for both the therapist and client to develop a positive relationship enhances the powerful effects of synchronization [126,132].

Emotional co-regulation is linked to synchronization of non-verbal behaviour [139,140]. Movement synchrony has been shown to aide emotional sharing, motor empathy, mutual understanding and adaptive emotional-regulation [136]. Therefore, the idea of having an embodied therapist seems to be attractive for the building of the therapeutic relationship. Embodiment techniques such as the mutual timing of responses including imitation, movement synchronization, eye gaze, breathing, posture, word use (content and function, tone/pitch) build the therapeutic alliance and thus can enhance affective co-regulation [136]. 

The IS model of psychotherapy has three levels of processing speed on three different time scales. The first and fastest level of the IS model involves a perceptual motor interbody coupling process that occurs within 10 s via movement synchrony (of face, eye gaze, breathing patterns, whole-body movements) that is beneficial for the therapeutic process. Interbody coupling then facilitates the next more complex social-cognitive level of processes constituting the alliance (occurring within 10 s–1 h). This includes the mutual sharing of subjective experiences involving common language and reasoning. These mutual sharing of experiences both build on the perceptual motor processes and are grounded in them. At the third level (taking several weeks or more to develop), affective co-regulation emerges which comprises of joint regulation of affective responses and their corresponding physiological correlates. Although emotional co-regulation can often occur automatically through synchronization of the client’s and therapist’s motor movements, it requires more than automatic physiological matching. An embodied therapist is required to explicitly use experiential dynamic techniques for co-regulation that either up or down regulates the client. By the therapist responding to the client in a varied way the client returns towards a homeostatic balance and the interaction is contained within healthy limits [136,141]. At the third processing level, there is differentiation between explicit and implicit emotional regulation for the client. For explicit emotional regulation to occur Koole and Tschacher [136] propose that it is mediated by common language (linguistic alignment, goal related language). However, the awareness of the three levels of process is not made explicit to the client in the IS model. 

This paper builds and further expands on the unconscious processes of the IS model to enhance the therapeutic alliance. A new idea is proposed that the explicit processes of imitation and movement synchronization be used in a conscious way between the therapist and client. The deliberate incorporation of synchronized specific emotional, facial, gestural and whole-body mirroring/imitation mechanisms including breathing patterns of the therapist with the client thus create a first emotional validation experience for the client in the Emotional Field. The client is given a foreshadowing of this process in psycho-education prior to stepping into the Emotional Field. Thus, imitation and movement synchronization is implemented as a shared tool in the Emotional Field to avoid rejection by some clients. The client is of course not mirrored continuously and is given a break from this process when they return to their chair in the cognitive position to reduce any self-consciousness, intensity or annoyance.

If the synchronization process is not made conscious to the client, some may react defensively when they become aware of the mirroring process in therapy. An embodied therapist should be well aware of his or her own interoceptive impulses and is always ‘on’ with the idea that they can also deliberately activate the client’s emotional regulation processes by down- regulating and moderating their own emotional mirroring responses via more moderated facial expressions, softer tones and slower breathing.

Peri et al. [142] explored the use of therapeutic embodied simulation and mirroring in helping clients with post-traumatic stress disorder to down regulate during exposure to trauma memories. In this process, the emotional responses of the client are transferred to the therapist through embodied simulation. The therapist experiences viscerally the client’s pain which is expressed in facial imitation. Then the therapist activates their own emotional and cognitive regulation processes, which moderate their own emotional response. This in turn is expressed to the client in a more neutral facial expression. These modulated emotional responses are mirrored back to the client, who is helped by experiencing painful emotions in a more modulated way, thus exerting some containment and control over distressing emotional responses.

However, when in the Emotional Field of our model, explicit embodiment techniques such as imitation and movement synchronization can have positive effects on the therapeutic relationship which can be viewed as a first tool for emotional validation and co-regulation. Our integrated model also includes explicit and conscious emotional acceptance and emotional mastery processes thereby engendering a permanent change between all three levels of processing/feedback loops as proposed in the IS model. We view an important integration of the IS levels of processing model into our switch model. Level 1 (Perceptual-motor processes) of the IS model could be depicted in our model by enhancing the therapeutic relationship unconsciously via movement synchrony that occurs during the mindfulness exercises, the selection of the problem person, the short imagination and the reactivation of the client’s relevant person, which is imitated by the therapist. Level 2 of the 

IS model (complex cognition) fits well into our Step Out (Neutral) position. Level 3 of the IS model (Explicit and Implicit Emotion Regulation) is similar to the Expert Position and in the working processes of the Emotional Field. 

## 6. Case Study

**Figure 4:**
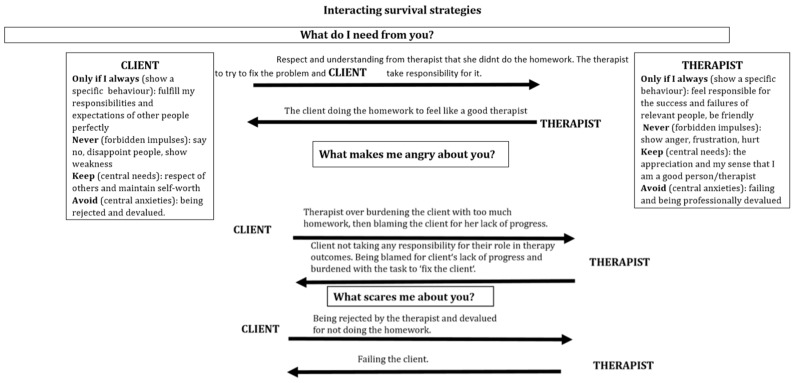
Hypothetical interacting survival strategies between a therapist and a client resulting in escalation.

**Figure 5:**
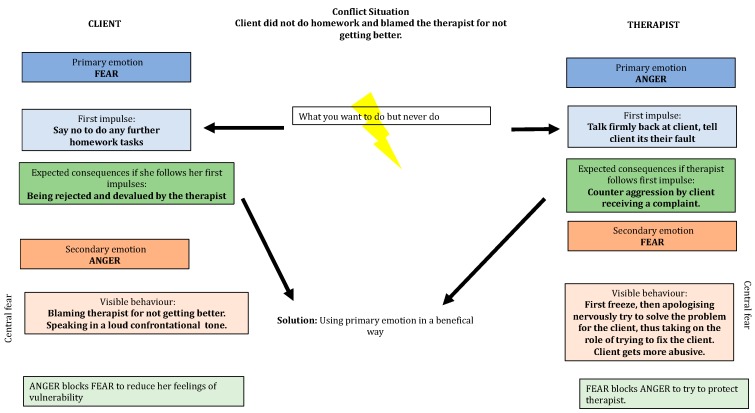
Interacting reaction chains, highlighting potential intervention points for change in the interaction.

A 38-year-old accountant comes for treatment suffering from exhaustion and depressive moods. She is fighting to keep not only her job but basically to keep the entire company going, because the company has moved into the red. After a colleague and her direct boss went on sick leave, she has been running the entire business practically alone. She does overtime daily because in general she feels responsible for everything and even on weekends cannot leave her work behind. She is suffering from lack of sleep and an inability to concentrate. In addition, she has been suffering from stomach and neck pain for many months. Often, she feels beaten down, sad and helpless, because no one supports her. She comes to the session not having completed her set homework from the session before and then angrily blames the therapist because she is not getting any better. A therapist unaware of their own and the client’s emotional survival strategy is likely to have a problematic outcome in this scenario as shown in Figure 4 and Figure 5 below.

As can be seen from Figure 4 the therapist has overlooked the fact that the client wants to be perfect in her role too and is showing the therapist anger because the client fears being imperfect and letting others down. The therapist is unware of the forbidden impulse of their own anger which is being blocked by fear. The therapist’s fear left unregulated is impulsively expressed in the interaction with the client as a kind of overly apologetic behaviour in response to their client’s anger. As the therapist succumbs to the unconscious processes of their own survival strategy the therapist tries to rescue the client by assuming full responsibility for the client’s lack of homework completion.

This is a problematic outcome for both the therapist and the client. First the client has not learnt to regulate her anger, nor to get in touch with her fear of being imperfect. The therapist has inadvertently reinforced the client’s need to be perfect by taking full responsibility for the client’s failure in the homework task. The therapist missed the opportunity to get in touch with the anger impulses in their own body in response to the clients’ criticisms.

### What Would an Embodied Therapist do in this Situation?

Being aware of and feeling the anger inside of the therapist is an opportunity for the therapist to engage in a self-soothing task of breathing slowly and using their own body and face to show a calm expression which could have also had the positive effect of down-regulating the client’s anger too. Once the anger is down-regulated the therapist could have been in a position to reflect on the function of their felt anger for the interaction focus with their client instead of blocking their anger. The therapist can use the mobilizing energy of their regulated anger with the aid of their body to express their frustration with the client. For example, the therapist could show positive surprise to the client’s lack of homework completion, leaning forwards towards the client in their chair with open arms and taking up a lot of space with their body to indicate power.

This would allow the client to feel some limits when expressing unregulated anger and feel some ownership over their fears of imperfection and still feel accepted by the therapist. The therapist then has an opportunity to check in with the client’s central fears within the therapeutic relationship and ask the client to show what their body wants to do now, how their body is feeling in the current moment. This gives the therapist an opportunity to synchronize her whole body with the client’s body expression of fear, giving the client a first type of emotional validation and acceptance of fear. The client has the opportunity to experience, that the central fear of rejection does not happen to them when they get in touch with their fear. Instead the client has an opportunity to feel bonded to and validated by the therapist. This in turn can benefit the client enormously in helping to break their survival strategy in their interaction with other significant people in their life outside of therapy. The stage is set for the client to use the positive therapeutic alliance to begin the process of accepting her imperfections in her work relationships and learn not to take on so much responsibility at work by setting limits and saying no.

## 7. Conclusions and Future Directions

We have examined an integrated model of psychotherapy for use with clinical populations in which bottom-up embodiment techniques are proposed to compliment the top-down method of CBT. Embodiment techniques such as movement synchrony and imitation processes readily and easily compliment and extend the ‘talking therapies’ as well as addressing some of their limitations. Our integrated switch model would gain profit from further empirical studies evaluating its effectiveness with a wide range of clinical populations to fully assess its validity. Although some preliminary case studies and group analysis based on our switch model are promising, randomised controlled trials are needed to further determine its efficacy and validity. The integration of the IS and our switch model stands to be a promising avenue for further research. It may be particularly pressing to begin to take standardized assessment measures of interpersonal synchrony between the client and therapist (both unconscious and conscious applications) such as movement synchrony, alliance and emotional regulation and to determine the predictive effects of these variables on reducing client psychopathology.
